# Missing not at random in end of life care studies: multiple imputation and sensitivity analysis on data from the ACTION study

**DOI:** 10.1186/s12874-020-01180-y

**Published:** 2021-01-09

**Authors:** Giulia Carreras, Guido Miccinesi, Andrew Wilcock, Nancy Preston, Daan Nieboer, Luc Deliens, Mogensm Groenvold, Urska Lunder, Agnes van der Heide, Michela Baccini, ACTION consortium, Agnes van der Heide, Ida J. Korfage, Judith A. C. Rietjens, Lea J. Jabbarian, Suzanne Polinder, Hans van Delden, Marijke Kars, Marieke Zwakman, Luc Deliens, Mariëtte N. Verkissen, Kim Eecloo, Kristof Faes, Kristian Pollock, Jane Seymour, Glenys Caswell, Andrew Wilcock, Louise Bramley, Sheila Payne, Nancy Preston, Lesley Dunleavy, Eleanor Sowerby, Guido Miccinesi, Francesco Bulli, Francesca Ingravallo, Giulia Carreras, Alessandro Toccafondi, Giuseppe Gorini, Urška Lunder, Branka Červ, Anja Simonič, Alenka Mimić, Hana Kodba-Čeh, Polona Ozbič, Mogens Groenvold, Caroline Arnfeldt, Anna Thit Johnsen

**Affiliations:** 1 Oncological Network, Prevention and Research Institute (ISPRO), Florence, Italy; 2 Department of Clinical Oncology, University of Nottingham, Nottingham, UK; 3 Lancaster University, International Observatory on end of life care, Lancaster, UK; 4 Department of Public Health, Erasmus University, Rotterdam, Netherlands; 5 Vrije Universiteit Brussel & Ghent University, Brussels, Belgium; 6 Department of Public Health, Copenhagen University, Copenhagen, Denmark; 7 University Clinic for Respiratory and Allergic Diseases, Golnik, Slovenia; 8 Department of Statistics, Computer Science, Applications ‘G. Parenti’ (DISIA), University of Florence, Florence, Italy

**Keywords:** Missing data, MAR, MNAR, Advance care planning, Oncology, Quality of life

## Abstract

**Background:**

Missing data are common in end-of-life care studies, but there is still relatively little exploration of which is the best method to deal with them, and, in particular, if the missing at random (MAR) assumption is valid or missing not at random (MNAR) mechanisms should be assumed. In this paper we investigated this issue through a sensitivity analysis within the ACTION study, a multicenter cluster randomized controlled trial testing advance care planning in patients with advanced lung or colorectal cancer.

**Methods:**

Multiple imputation procedures under MAR and MNAR assumptions were implemented. Possible violation of the MAR assumption was addressed with reference to variables measuring quality of life and symptoms. The MNAR model assumed that patients with worse health were more likely to have missing questionnaires, making a distinction between single missing items, which were assumed to satisfy the MAR assumption, and missing values due to completely missing questionnaire for which a MNAR mechanism was hypothesized. We explored the sensitivity to possible departures from MAR on gender differences between key indicators and on simple correlations.

**Results:**

Up to 39% of follow-up data were missing. Results under MAR reflected that missingness was related to poorer health status. Correlations between variables, although very small, changed according to the imputation method, as well as the differences in scores by gender, indicating a certain sensitivity of the results to the violation of the MAR assumption.

**Conclusions:**

The findings confirmed the importance of undertaking this kind of analysis in end-of-life care studies.

**Supplementary Information:**

The online version contains supplementary material available at 10.1186/s12874-020-01180-y.

## Background

Missing data are common in palliative and end-of-life care studies, where 20–50% of participants withdraw early, mostly because of deterioration and/or death [1]. A systematic review and meta-analysis of randomized controlled palliative intervention trials found about one quarter of primary endpoint data missing [2]. In the literature, several methods have been proposed for dealing with missing data, but there has been relatively little exploration of which is best in the palliative care setting [3]. The appropriateness of the chosen method is strongly related to the nature of the mechanism generating missing data. Missing data can be categorized as: missing completely at random (MCAR), when the probability of an observation being missing does not depend on both observed and unobserved data; missing at random (MAR), when the probability of an observation being missing depends only on the observed; and missing not at random (MNAR), when the probability of an observation being missing also depends on unobserved data [4].

Complete-case analysis, that consists in performing the analysis on the subset of subjects with complete information, was the most common approach to treat missing data in randomized clinical trials as recently as 2013 [5, 6]. However, unless the missingness mechanism is MCAR, this may lead to biased results. When the MCAR assumption is not valid, alternative strategies can be adopted to deal with missing data: inverse probability weighting, doubly robust inverse probability weighting, maximum likelihood estimation, multiple imputation (MI). Among them, MI is widely recognised as the most appropriate one in many fields [7]. MI creates several complete versions of the data by replacing each missing value with more than one plausible value. Each of the resulting complete datasets is then analyzed with standard statistical methods and the results pooled for final inference using the Rubin’s combination rule, to obtain a point estimate and a measure of precision which accounts for uncertainty due to missing information [4, 8]. There are several ways to implement MI that could be run under MAR and MNAR [9–12]. One of these is the Multivariate Imputation by Chained Equations (MICE) which relies on the MAR assumption [9, 10], but can be modified in order to account for MNAR mechanisms [13].

Missing data in end-of-life care studies can arise due to the fact that one or more questionnaire items are missing, or due to the fact that all the questionnaire items are missing (missing form). While missing items may be due to simple omissions in questionnaire compilation, reasons for missing a whole questionnaire may relate to sudden change in the patient’s health status or to the patient’s sensitivity to specific issues which could be not adequately measured by the collected variables. In these cases, the missingness mechanism may be MNAR [14–18]. Although it is known that performing MI under the assumption of MAR when the actual missingness mechanism is MNAR may produce biased estimates [4], this issue is not widely appreciated in dealing with missing data in palliative care studies [3]. Even if it is not possible to distinguish between MNAR and MAR patterns using observed data, the robustness of the MAR assumption can be investigated through sensitivity analyses [4, 14]: if the results obtained under MAR and specific MNAR assumptions are similar, one can conclude that the presence of unobserved factors does not affect the conclusions.

The aim of this study was to compare the performance of different MI methods within the ACTION (Advance care planning – an innovative palliative care intervention to improve quality of life in oncology) study by treating separately missing items and missing questionnaires [15]. Using a preliminary dataset, we handled the missing data by applying a MICE procedure under the standard assumption of MAR and also under MNAR by using a pattern mixture-model approach, [4, 16] which distinguished between missing values due to missing items and missing the whole questionnaires. We focused on target analyses to evaluate the sensitivity of the results to the use of the two procedures.

## Methods

### Data

The ACTION study is a phase III multicenter cluster randomised controlled trial, following the CONSORT guidelines, on the effects of advance care planning (ACP) in patients with advanced lung or colorectal cancer. The ACTION Respecting Choices ACP intervention involves trained healthcare professionals who assist patients and their relatives in reflecting on the patient’s goals, values and beliefs and in discussing their healthcare wishes. The intervention has the potential to improve current and future healthcare decision making, provide patients with a sense of control, and improve quality of life. In total, 22 hospitals in 6 countries were randomised to be intervention or control sites, with up to 1360 patients participating. At the intervention sites, patients are offered interviews with a trained ACP facilitator, whereas in the control sites, patients receive usual care. All participating patients are asked to complete questionnaires at baseline, and again after 2.5 and 4.5 months; the questionnaires assess quality of life, and the extent to which care as received is aligned with their preferences, their evaluation of decision-making processes and quality of end-of-life care (see additional file 2) [15].

Ethical approval was obtained from the Research Ethics Committee (REC) of the coordinating centre (‘Medische Ethische Toetsings Commissie Erasmus MC’), as well as RECs in all participating countries. The trial was registered in the International Clinical Trials Registry Platform (ISRCTN63110516) per 10/3/2014.

Within the ACTION study it was decided to perform a preliminary analysis on a first subsample of the enrolled subjects, with the aim to explore methods for dealing missing data. Our analysis was based on the records of 487 patients, representing the 36% of the final expected sample, containing information collected through questionnaires at baseline and at 2.5 months, for a total of 121 variables. Baseline data included personal information (gender, age, marital status, living with a partner or alone, living in a private household or in an institution/care facility, children, years of education, religiosity, ethnic group), information on diagnosis (small cell or non-small cell lung cancer, colon cancer, rectal cancer), cancer stage (III or IV lung cancer; metachronous metastases or IV colorectal cancer), WHO performance status (a measure of how well a person with cancer is able to carry on ordinary daily activities), and current treatment (chemotherapy, radiation therapy, immunotherapy, targeted therapy). Using baseline and follow-up questionnaires, we calculated scores for quality of life and symptoms (primary endpoints) and for shared decision-making, satisfaction with care and coping with illness (secondary endpoints) (Table 1). All the scores, except that measuring patient involvement which was built ad hoc for the present study, are validated and largely used in the context of psychometrics [17–22]. They are continuous variables ranging between 0 and 100. Since an intermediate analysis on the treatment effect was not planned by protocol, this preliminary analysis was blinded in respect to treatment arm and country of the participants, as well as in respect to their survival. Due to the fact that few patients are expected to die during the first 2.5 months of follow up, for sake of simplicity, in our analysis we assumed that all patients were still alive at the completion of the second questionnaire, meaning that no form was missing because of patient’s death (see discussion).

**Table 1:**
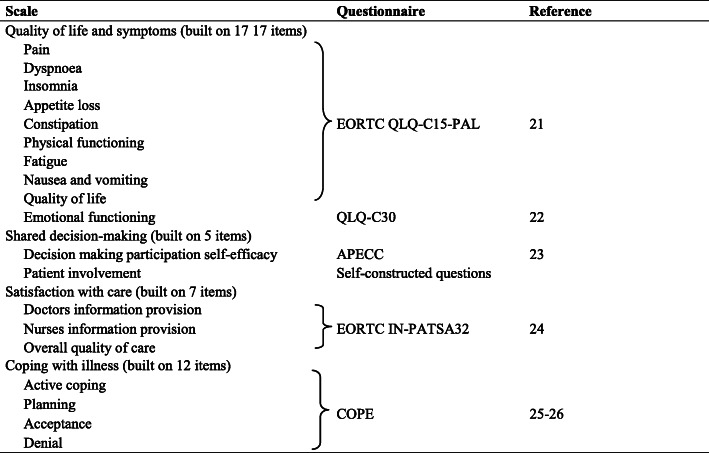
Primary and secondary endpoints (scales) measured with questionnaires at baseline and follow-up

### Statistical methods

In our analysis we first applied the MICE procedure under MAR assumption. The procedure was applied to all the variables with missing data, including variables on personal information, and on diagnosis and treatments. Then we performed a sensitivity analysis assuming alternative MNAR mechanisms, within a pattern mixture-model approach [4, 16]. MI under MNAR required the specification of additional assumptions on the missingness mechanism and the modification of the MICE algorithm.

According to the main purpose of the ACTION study, the sensitivity analysis focused on relevant outcomes of quality of life and symptoms scores. We estimated means and 90% confidence intervals of these outcomes on the overall sample and by gender and their correlations with other scores (i.e. shared decision making, satisfaction with care and coping with illness), under the different MI approaches. Differences among MI procedures were evaluated by comparing point estimates and their confidence intervals in a descriptive way. If, under all the MNAR models, the direction of the explored relationships was the same as that under MAR and the confidence intervals largely overlapped, we concluded that the results were robust to violation of the MAR assumption. Due to the fact that at this stage of the analysis we are not interested in performing statistical tests, but only in describing the results, we preferred to report the 90% confidence intervals instead of the 95% ones in order to discourage their use as a surrogate of *p*-value = 0.05 [23].

### Multiple imputation by chained equations under MAR assumption

Let **Y** = (*Y*_*1*_*,…,Y*_*J*_) be the *n*⨯*J* matrix of the data, where *Y*_*j*_ *= (y*_*1j*_*, y*_*2j*_*,…y*_*nj*_*)*^T^ is the vector of length *n* representing the values assumed by the *j*th variable in the *n* subjects. Let **R** = (*R*_*1*_*,…, R*_*J*_) be a *n*⨯*J* matrix, with *R*_*j*_ = (*r*_*1j*_, *r*_*2j*_, …,*r*_*nj*_)^T^ vector of the missingness indicators for the *j*th variable (*r*_*ij*_ = 0 if *y*_*ij*_ is missing and *r*_*ij*_ = 1 otherwise). Let us denote the observed entries and the missing entries of **Y** as **Y**_obs_ and **Y**_mis_, respectively. Analogously, let *Y*_*j *obs_ and *Y*_*j *mis_ be the observed and the missing entries of the vector *Y*_*j*_. Under the MAR assumption of conditional independence between **Y**_mis_ and **R** given the observed data **Y**_obs_, ƒ(**Y**_mis_|**Y**_obs_, **R**) = ƒ (**Y**_mis_|**Y**_obs_), the MICE algorithm required the specification of the univariate conditional distributions *f(Y*_*j*_*|Y*_*-j*_*,* ***θ***_***j***_*)* in the form of regression models for the variables with missing values, where *Y*_*-j*_ *= (Y*_*1*_*, …, Y*_*j-1*_*, Y*_*j + 1*_*, …, Y*_*J*_*)* and ***θ***_***j***_ was a vector of unknown regression parameters [9]. In our analysis, in order to avoid collinearity and related computational problems, assuring at the same time good prediction, a preliminary selection of the predictors to be included in the regressions was performed (in practice some of the elements of ***θ***_*j*_ have been set to zero). In particular, we regressed each variable *Y*_*j*_ with missing entries on each of the other variables, completed by a preliminary imputation which draw from their empirical marginal distribution. Then, we calculated the Akaike’s information criterion (AIC) for each regression and selected as predictors for *Y*_*j*_ the 15 variables which lead to the smallest AIC values [24]. Considering the number of observations and the large percentage of missing values in most of the follow up scores, 15 was considered an appropriate number of covariates [10]. The marginal regressions used in this preliminary procedure and in the following imputation algorithms varied according to the nature of the outcome variable (linear regression for continuous variables, logistic regression for factor variables with 2 levels, multinomial logistic regression for factor variables with more than 2 levels, and proportional odds models for ordered variables). Finally, for each incomplete variable, the set of the selected predictors was enriched by including the indicators of gender, age, type of cancer and WHO performance status. The selected predictors for each incomplete variable are reported in Figure A1 of the additional file 1.

Once defined the conditional distributions *f(Y*_*j*_*|Y*_*-j*_*,* ***θ***_***j***_*)* and assumed non-informative priors on ***θ***_***j***_, the MICE algorithm consisted in the following steps. First, we randomly drew an initial imputation ${\hat{Y}}_1,\dots, {\hat{Y}}_J$ for the missing values in **Y**_mis_, by sampling from the empirical marginal distributions of the variables with missing entries (*step 0*). Then, for the first variable *Y*_*j*_ with missing entries:

1. we sampled ${\hat{\theta}}_j$ from the posterior distribution $f\left({\theta}_j|{Y}_{j\ \mathrm{obs}},{\hat{Y}}_{-j}\right)$,
2. we drew ${\hat{Y}}_{j\ \mathrm{mis}}$ from the posterior predictive distribution $f\left({Y}_j|{Y}_{j\ \mathrm{obs}},{\hat{Y}}_{-j},{\hat{\theta}}_j\right)$.

We repeated *steps 1 *and *2* sequentially for each variable with missing entries in the dataset, and we repeated the entire procedure, excluding *step 0*, until algorithm convergence (100 iterations) [25]. At the end, we got a complete dataset. We created several complete versions of the data by  repeating the procedure M times, choosing *M* according to the rule of thumb based on the average percentage rate of missingness [26].

The MICE procedure was implemented by using the mice library of the R software [27, 28].

### Multiple imputation by chained equations under MNAR assumption

Let us suppose that the MAR assumption is not valid. This, in general, implies that the posterior predictive distribution of *Y*_*j* mis_ at step 2 of the MICE algorithm depends on **R**. In order to account for this dependency, according to the pattern mixture-model approach [4, 16], the MICE algorithm can be modified defining distinct posterior predictive distributions depending on the missing data patterns in **R**. For example, let us assume that we are in the simple case in which the value of *Y*_*j* mis_ depends on the observed and on the fact that *Y*_*j*_ is missing, but not on the fact that other variables are missing. In this case, we would have two distinct posterior predictive distributions, one for *R*_*j*_ = 0 and one for *R*_*j*_ = 1:

$$ f\left({Y}_j|{Y}_{j\ \mathrm{obs}},{\hat{Y}}_{-j},{{\hat{\theta}}_j}^{R_j=0}\right) $$ and $$ f\left({Y}_j|{Y}_{j\ \mathrm{obs}},{\hat{Y}}_{-j},{{\hat{\theta}}_j}^{R_j=1}\right) $$,

and the step 2 of the MICE algorithm can be modified as follows. After having sampled ${{\hat{\theta}}_j}^{R_j=1}$ from the posterior distribution $f\left({\theta}_j|{Y}_{j\ \mathrm{obs}},{\hat{Y}}_{-j}\right)$, we generate ${{\hat{\theta}}_j}^{R_j=0}$ from the conditional distribution $f\left({\theta}_j^{R_j=0}|{\hat{Y}}_{-j},{\hat{\theta}}_j^{R_j=1}\right)$, a priori defined according to an assumed hypothesis on the MNAR mechanism. Finally, we sample ${\hat{Y}}_{j\ \mathrm{mis}}$ from the posterior predictive distribution $f\left({Y}_j|{Y}_{j\ \mathrm{obs}},{\hat{Y}}_{-j},{{\hat{\theta}}_j}^{R_j=0}\right)$. More in general, when the value of *Y*_*j* mis_ depends also on the fact that other variables are missing, there are in principle distinct predictive distributions for each pattern of missing data [29].

### Partition of the missing values and modified MICE in the ACTION study

In our analysis, we explored the violation of the MAR assumption, making a distinction between missing items due to the fact that the patient did not reply to some items in a questionnaire and missing items due to the fact that the form was completely missing (this could happen for the second questionnaire, but not for the baseline one), for which a violation of the MAR assumption could be hypothesised. In particular, we introduced two matrices of missingness indicators, **R**^**I**^ which indicated the missing values of the first type and **R**^**F**^ which indicated the missing values of the second type. The two matrices described a partition of the missing values; for simplicity we called **M**^**I**^ the first element of the partition (collection of the missing data defined by **R**^**I**^) and **M**^**F**^ (collection of missing data defined by **R**^**F**^). Then, we assumed a possible MNAR mechanism for the missing values in **M**^**F**^:

$$ f\left({\mathbf{Y}}_{\mathrm{mis}}|{\mathbf{Y}}_{\mathrm{obs}},{\mathbf{R}}^{\mathbf{I}},{\mathbf{R}}^{\mathbf{F}}\right)=f\left({\mathbf{Y}}_{\mathrm{mis}}|{\mathbf{Y}}_{\mathrm{obs}},{\mathbf{R}}^{\mathbf{F}}\right), $$

with ƒ (**Y**_mis_|**Y**_obs_, **R**^**F**^) possibly different from ƒ (**Y**_mis_|**Y**_obs_).

Two distinct posterior predictive distributions were defined for each *Y*_*j*_ with missing values in **M**^**F**^, one for *R*^*F*^_j_ = 0 and one for *R*^*F*^_j_ = 1:

$$ f\left({Y}_j|{Y}_{j\mathrm{obs}},{\hat{Y}}_{-j},{{\hat{\theta}}_j}^{R_j^F=0}\right) $$ and $$ f\left({Y}_j|{Y}_{j\mathrm{obs}},{\hat{Y}}_{-j},{{\hat{\theta}}_j}^{R_j^F=1}\right). $$

The described violation of the MAR assumption required a modification of the MICE algorithm that relied on a model for $f\left({\theta}_j^{R_j^F=0}\left|{\hat{Y}}_{-j},{\hat{\theta}}_j^{R_j^F=1}\right.\right)$. The modified MICE algorithm that we implemented for our analysis was the following. After having randomly drawn an initial imputation ${\hat{Y}}_1,\dots, {\hat{Y}}_J$ for the missing values by sampling from the empirical marginal distributions of the variables with missing entries (*step 0*), we imputed the missing values for the first variable *Y*_*j*_ with missing entries, according to the following steps:

1. we sampled ${\hat{\theta}}_j$ from the posterior distribution $f\left({\theta}_j|{Y}_{j\ \mathrm{obs}},{\hat{Y}}_{-j}\right)$,
2. we drew ${\hat{Y}}_{j\ \mathrm{mis}}$ from the posterior predictive distribution $f\left({Y}_j|{Y}_{j\ \mathrm{obs}},{\hat{Y}}_{-j},{\hat{\theta}}_j\right)$,
3. if *Y*_*j*_ had missing entries in **M**^**F**^, we generated ${\hat{\theta}}_j^{R_j^F=0}$ from the conditional distribution $f\left({\theta}_j^{R_j^F=0}|{\hat{Y}}_{-j},{\hat{\theta}}_j^{R_j^F=1}\right)$, a priori defined according to our hypothesis on the MNAR mechanism (see below), and we sampled ${\hat{Y}}_{j\ \mathrm{mis}}$ from the posterior predictive distribution $f\left({Y}_j|{Y}_{j\  obs},{\hat{Y}}_{-j},{\hat{\theta}}_j^{R_j^F=0}\right)$.

We repeated *step 1*-*step 3* for all variables with missing entries sequentially, until algorithm convergence (100 iterations). We repeated the whole procedure *M* times in order to create *M* complete versions of the data set. In running the algorithm, we ordered the variables according the number of missing values (from the variable with fewer missing entries to that one with more missing entries).

### Assumptions on the MNAR mechanism

We assumed that the MAR assumption was violated with reference to the six primary endpoints expressing the patient’s health status, as measured by the second questionnaire: follow-up scores pain (PA), dyspnoea (DY), emotional functioning (EF), physical functioning (PF), fatigue (FA) and quality of life (QOL). Under the general assumption that reasons for missing the second questionnaire as a whole may relate to sudden changes in the patient’s health status or to sensitivity to specific issues not adequately measured by the other variables [4, 14, 30–33], patients with a poorer health status were those for whom we expected to have larger probability of missing form. Our definition of the MNAR mechanisms reflected these hypotheses.

Even if in principle the model for $f\left({\theta}_j^{R_j^F=0}\left|{\hat{Y}}_{-j},{\hat{\theta}}_j^{R_j^F=1}\right.\right)$ could be highly complex, we specified two simple alternative models [13, 34, 35]. The first model assumed a constant shift *k* in the expected value of each of the six variables between observed and missing observations belonging to **M**^**F**^. We considered four different shifts *k*_*1*_*, …, k*_*4*_ defined as the equispaced values between 0 and ½ interquartile range of the variable. The second model allowed the shift *k* to vary among individuals according to their WHO performance status. Let *Y*_*WHO*_ be the variable expressing the WHO performance status, and *sd*(*Y*_*j* obs_) the standard error for one of the six primary endpoints, calculated on the observed values; the shift *k* was defined as *k* = *Y*_WHO_⨯*sd*(*Y*_*j*obs_)/4. The shift was always assumed to be in the direction of worsening the patient’s health conditions (subtracted for QOL, PF, EF and added for the other scores).

### Analysis of data

We implemented the algorithm for the MNAR model by modifying the existing mice function of the mice R library. We used the passive imputation built-in method in order to incorporate at each step of the imputation algorithm the transformations required by the MNAR assumptions [27, 28, 34].

At the end of the MI procedure (MICE or modified MICE), each of the *M* complete datasets was analysed applying standard statistical methods and the results were combined according to the Rubin’s rules [4]. Indicating with *Q* the unknown parameter of interest, for example the average quality of life score, let ${\hat{Q}}_m$ be the point estimate of *Q* and ${\hat{U}}_m$ the estimate of its variance, arising from the analysis on the *m*th dataset (*m* = 1, …, *M*). The combined estimate of *Q* was equal to $\overline{Q}=\sum \limits_{m=1}^M\frac{{\hat{Q}}_m}{M}$ and its estimated variance was *T* = *U*+(1 + 1/*M*) *B*, where $U=\sum \limits_{m=1}^M\frac{{\hat{U}}_m}{M}$ and $B=\frac{\sum_{m=1}^M{\left({\hat{Q}}_m-\overline{Q}\right)}^2}{\left(M-1\right)}.$

## Results

In Tables 2 and 3, we report descriptive statistics and percentage of missing values for socio-demographic, diagnosis and treatment variables measured at baseline, and for baseline and follow-up scores of quality of life and symptoms, shared decision-making, satisfaction with care and coping with illness. Between 1 and 15% of socio-demographic data, clinical data and baseline scores were missing. Missing values at follow-up were around 36–39%, mostly due to missing forms (170/487; 35%).

**Table 2:** Descriptive statistics on personal information and diagnosis information at baseline

Personal information					
Age		Live with a partner, **n (%)**		Religious, n(%)	
missing, n (%)	5 (1.03)	missing	11 (2.26)	missing	13 (2.67)
mean (sd)	65.99 (9.90)	yes	340 (69.82)	yes	225 (46.20)
**Gender**, n (%)		no	136 (27.93)	no	185 (37.99)
missing	4 (0.82)	**Living place**, n (%)		prefer not to specify	64 (13.14)
male	292 (59.96)	missing	14 (2.87)	**Minority ethnic group**, n (%)	
female	191 (39.22)	private household	447 (91.79)	missing	20 (4.11)
**Marital status**, n (%)		institution/care facility	3 (0.62)	yes	7 (1.44)
missing	8 (1.64)	other	23 (4.72)	no	460 (94.46)
married	324 (66.53)	**Children**, n (%)		**Years of education**	
unmarried	41 (8.42)	missing	8 (1.64)	missing, n (%)	73 (14.99)
divorced/separed	61 (12.53)	yes	414 (85.01)	mean (sd)	13.39 (4.74)
widowed	53 (10.88)	no	65 (13.35)		
**Diagnosis information**					
**Type of cancer**, n (%)		**WHO performance status**, n (%)		**Stage of cancer**, n (%)	
missing	4 (0.82)	missing	5 (1.03)	missing	7 (1.44)
small cell lung	62 (12.73)	0 fully active	167 (34.29)	III lung	59 (12.11)
non-small cell lung	218 (44.76)	1 symptomatic but completely ambulatory	255 (52.36)	IV lung	224 (46.00)
colon	155 (31.83)	2 symptomatic, < 50% in bed during the day	58 (11.91)	IV colorectal	134 (27.52)
rectal	48 (9.86)	3 symptomatic, > 50% in bed, but not bedbound	2 (0.41)	metachronous metast colorectal cancer	63 (12.94)
**Current treatment**					
**Chemotherapy**, n (%)		**Immunotherapy**, n (%)			
missing	0	missing	0		
no	119 (24.44)	no	450 (92.40)		
yes	368 (75.56)	yes	37 (7.60)		
**Radiation therapy**, n (%)		**Targeted therapy**, n (%)			
missing	0	missing	0		
no	449 (92.20)	no	428 (87.89)		
yes	38 (7.80)	yes	59 (12.11)

**Table 3:** Means of the scores (calculated using observed data) at baseline and at 2.5 months of follow-up (sd: standard deviation)

Variable	Baseline		Follow-up	
	mean (sd)	missing (%)	mean (sd)	missing (%)
Pain	21.41 (24.03)	6 (1.23)	20.67 (25.16)	175 (35.93)
Dyspnoea	25.28 (28.08)	7 (1.44)	26.33 (27.83)	173 (35.52)
Insomnia	27.36 (28.03)	7 (1.44)	25.27 (26.35)	173 (35.52)
Appetite loss	21.14 (29.95)	6(1.23)	21.87 (29.24)	173 (35.52)
Constipation	19.72 (29.14)	7(1.44)	18.33 (26.84)	176 (36.14)
Emotional functioning	78.41 (20.50)	11(2.26)	79.84 (21.22)	179(36.76)
Physical functioning	69.58 (23.91)	9 (1.85)	68.69 (25.60)	177 (36.34)
Fatigue	41.66 (26.22)	9 (1.85)	41.68 (25.70)	174 (35.73)
Nausea and vomiting	11.54 (20.42)	6 (1.23)	11.52 (22.39)	173 (35.52)
Quality of life	65.05 (20.27)	13 (2.67)	64.16 (21.84)	174 (35.73)
Decision making participation self-efficacy	75.06 (23.77)	17 (3.49)	76.53 (22.59)	179 (36.76)
Patient involvement	74.71 (18.24)	19 (3.90)	75.80 (18.30)	181 (37.17)
Doctors information provision	72.37 (23.74)	8 (1.64)	70.67 (24.41)	181 (37.17)
Nurses information provision	71.82 (24.29)	21(4.31)	70.05 (23.99)	191 (39.22)
Overall quality of care	76.21 (21.48)	12 (2.46)	73.03 (22.16)	183 (37.58)
Active coping	71.34 (25.49)	20(4.11)	68.26(27.40)	184 (37.78)
Planning	66.35 (27.97)	17 (3.49)	66.17 (27.92)	185 (37.99)
Acceptance	74.34 (21.70)	21 (4.31)	73.58 (22.34)	182(37.37)
Denial	23.20 (27.04)	25 (5.13)	20.68(25.37)	186 (38.19)

In the additional file 1, we reported the missing data pattern (figure A1), as well as the selected predictors for each univariate conditional regression used in the imputation procedures (figure A2).

Table 4 shows means and 90% confidence intervals of quality of life scores and symptoms at follow up, calculated from the observed data and after MI under MAR and MNAR assumptions setting *M* = 40, which approximately corresponded to the maximum percentage rate of missingness [26]. When compared with the values calculated on the observed data, the results under MAR suggested that missingness was related to poor health status and lower quality of life, with the means after MICE moving in the direction of a worse health, i.e. decreasing for QOL, PF, EF and increasing for PA, DY and FA. Assuming MNAR mechanisms the discrepancy between imputed values and observed values was markedly greater.

**Table 4:** Mean (90% confidence interval) of the scores at 2.5 months of follow-up up (Quality of life; Pain; Dyspnoea; Emotional functioning; Physical functioning; Fatigue) observed and imputed under the MAR assumption and using different models for departure from MAR (k_WHO_; k_1_; k_2_; k_3_; k_4_)

Variable	observed	MAR	k_**WHO**_	k_**1**_	k_**2**_	k_**3**_	k_**4**_
Quality of life	64.16 (64.09,64.24)	63.02 (62.94,63.09)	58.82 (58.74,58.90)	57.12 (57.04,57.19)	58.63 (58.56,58.71)	59.97 (59.89,60.04)	61.65 (61.57,61.72)
Pain	20.67 (20.59,20.76)	22.37 (22.28,22.46)	28.03 (27.92,28.14)	34.17 (34.04,34.30)	30.41 (30.29,30.52)	27.51 (27.40,27.61)	24.93 (24.83,25.02)
Dyspnoea	26.33 (26.23,26.42)	27.03 (26.94,27.12)	36.99 (36.86,37.12)	44.22 (44.07,44.37)	38.64 (38.51,38.78)	34.00 (33.89,34.12)	30.20 (30.09,30.30)
Emotional functioning	79.84 (79.77,79.91)	78.62 (78.54,78.69)	73.55 (73.46,73.64)	66.21 (66.09,66.33)	70.11 (70.01,70.21)	73.70 (73.61,73.79)	76.31 (76.22,76.39)
Physical functioning	68.69 (68.60,68.77)	67.24 (67.15,67.33)	62.35 (62.26,62.44)	59.94 (59.85,60.04)	61.60 (61.51,61.69)	63.23 (63.14,63.32)	65.43 (65.34,65.51)
Fatigue	41.68 (41.59,41.76)	42.90 (42.82,42.99)	49.62 (49.52,49.72)	51.14 (51.04,51.25)	48.91 (48.81,49.01)	46.83 (46.73,46.92)	44.75 (44.66,44.84)

Table 5 shows the mean scores by gender, calculated on the observed, under the MAR model and under the MNAR model that assumed a degree of departure from MAR dependent on the WHO performance status. Under both imputation approaches, there was a clear difference between genders, with males reporting a better health state than females. The size of the difference between genders increased under the MNAR model.

**Table 5:** Mean (90% confidence interval) of the scores at 2.5 months of follow-up (Quality of life; Pain; Dyspnoea; Emotional functioning; Physical functioning; Fatigue) for males and females observed and imputed under the MAR assumption and under the MNAR model assuming a shift depending on the WHO score (k_WHO_)

	observed		MAR		k_**WHO**_	
Variable	males	females	males	females	males	Females
Quality of life	66.67 (66.60,66.74)	60.06 (59.98,60.13)	65.69 (65.62,65.76)	58.94 (58.87,59.02)	61.32 *(*61.25,61.39*)*	55.01 (54.93,55.08)
Pain	19.22 (19.15,19.30)	22.92 (22.82,23.01)	20.71 (20.62,20.80)	24.90 (24.82,24.99)	26.01 *(*25.91,26.10*)*	31.12 (31.01,31.24)
Dyspnoea	25.04 (24.95,25.14)	28.69 (28.59,28.79)	25.75 (25.66,25.84)	29.00 (28.90,29.09)	35.34 *(*35.24,35.44*)*	39.50 (39.36,39.64)
Emotional functioning	83.96 (84.02,83.90)	73.02 (73.10,72.94)	82.88 (82.81,82.95)	72.11 (72.04,72.19)	78.64 (78.57,78.71)	65.80 (65.70,65.89)
Physical functioning	69.86 (69.77,69.95)	66.50 (66.41,66.58)	68.82 (68.74,68.90)	64.83 (64.74,64.93)	63.90 *(*63.82*,*63.98)	59.99 (59.90,60.09)
Fatigue	39.09 (39.01,39.18)	46.19 (46.10,46.28)	40.14 (40.05,40.22)	47.12 (47.04,47.21)	46.54 *(*46.45*,*46.63)	54.33 (54.22,54.43)

**Fig. 1:**
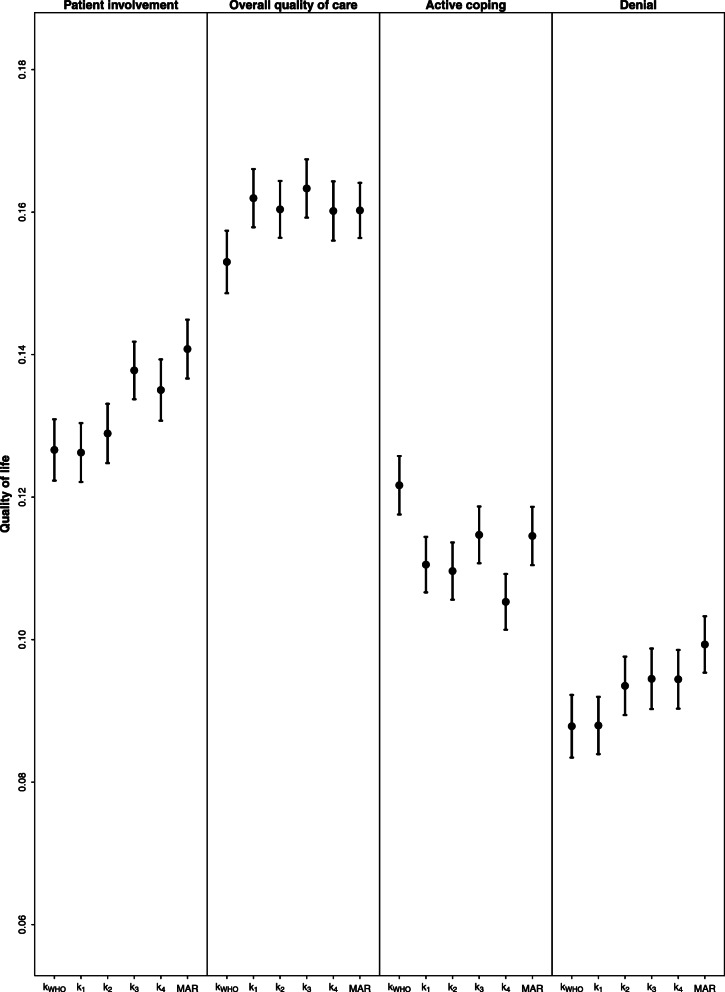
Correlations (90% confidence intervals) between Quality of life and secondary endpoints (Patient involvement; Overall quality of care; Active coping; Denial) calculated after MI under the MAR assumption and under different MNAR models (*k*_*WHO*_*; k*_*1*_*; k*_*2*_*; k*_*3*_*; k*_*4*_*)*

All correlations of secondary endpoints (patient involvement, overall quality of care, active coping, denial) with primary endpoints related to symptoms (FA, PA, DY) were negative, whereas those with primary endpoints related to functioning (QOL, PF, EF) were positive, although very low. As an example, in Fig. 1 we show the correlation coefficients between QOL and the four selected secondary endpoints. Overall quality of care showed the higher correlations with primary endpoints. Although in general correlations after MICE were robust to violations of the MAR assumption, sometimes a certain discrepancy was observed between the different imputation methods (see additional file 1 Figures A3-A7). In particular, under the MNAR scenarios all correlations involving denial were weaker than under MAR. A similar behaviour was observed also for patient involvement.

## Discussion

Reviews of randomized controlled trials indicate wide variation in how missing data are dealt with. Relatively few studies use MI (8%), and, when carried out, it is mainly under MAR assumption; few studies assume MNAR mechanisms [5, 6, 36]. As usual in end-of-life care studies, the proportion of missing values in the subset of the ACTION study data analysed in the present paper was high, particularly in relation to quality of life and symptoms outcomes. The percentage of missing forms at follow up was around 37%, thus higher than the 23% reported in a recent meta-analysis of randomized controlled trials on palliative interventions [2].

Although the availability of many variables should make the MAR assumption reasonable, quality of life is a multidimensional and complex concept and it is difficult to exclude the presence of relevant unmeasured factors, particularly in end of life care studies where reasons for missing values may relate to the patient’s health status or their sensitivity to specific issues [14, 30–33]. Because the violation of the MAR assumption can significantly affect final results, particularly when the proportion of missing values is high, we performed a sensitivity analysis to evaluate if the results obtained assuming MAR were robust to the presence of MNAR. In particular, we focused on means and correlations between primary and secondary outcomes or between primary outcomes and individual characteristics (gender).

Performing MI under both MAR and MNAR assumptions produced different estimates of the average score of the primary outcomes. The results under MAR suggested that missingness was related to poor health status and lower quality of life. This is consistent with the common sense idea that more critical patients could have greater problems in filling questionnaires. As expected, assuming MNAR mechanisms which explicitly assigned higher probabilities of missing form to those patients who experienced worse health status, the discrepancy between imputed values and observed values became markedly greater. Males showed a better health than females. After imputation, the difference became even more marked, particularly under the MNAR models. The marginal correlations between primary and secondary endpoints were consistently negative for symptoms and positive for functioning and quality of life. As an example, quality of life was positively associated with patient involvement, overall quality of care, active coping and denial. On the contrary, pain was negatively correlated with the same variables. In agreement with clinical expectations, overall quality of care had the strongest correlations with the primary outcomes. The correlations of the primary outcomes with denial appeared to be weaker under the MNAR scenarios than when assuming MAR. These findings suggest that the provisional indicators which we focused on in the present analysis were not always robust to violation of the MAR assumption.

The peculiar features of our approach are mainly two. First, we integrated the MNAR model within the MICE algorithm as implemented in the mice function through the passive imputation built-in method [34], so that imputation error was correctly propagated [37]. This is different from following a two-step procedure that first imputes each outcome under the MAR assumption, and then modifies the imputed values according to a specific model for MNAR [13, 31, 33, 38, 39]. Additionally, our algorithm accounts for the different nature of the missing values in the data set: missing entries satisfying the MAR assumption (15.3% of the total number of missing values) and missing entries, due to missing form, for which a MNAR mechanism could be hypothesised (84.7%).

In the literature, several algorithms have been proposed that modify MICE accounting for the violation of the MAR assumption [13, 29, 34, 35, 38]. Recently, Tompsett and colleagues [29] proposed an approach, called Not-At-Random Fully Conditional Specification (NARFCS), which generalizes the MICE algorithm to MNAR, by including in each univariate conditional regression model the missingness indicator of the variable to be imputed (the coefficient of which cannot be estimated from the data and is the object of the sensitivity analysis), as well as the missingness indicators of the other incomplete variables. In this way, it is possible to allow for the presence of correlations between each variable and the missingness indicators of the others. Unlike NARFCS, our approach assumed that these correlations were equal to zero, because it included a missing form indicator only in the univariate conditional models used for the imputation of quality of life and symptoms scores (i.e. the variable interested by MNAR). However, in a sensitivity analysis, we included the missing form indicator in each univariate conditional distribution, thus allowing for the possible correlation between having a missing form and all the variables in the data set. The results of this sensitivity analysis were very similar to those reported in the paper (see additional file 1 Tables A1-A2).

Recently, imputation algorithms based on nonlinear models, such as classification and regression trees or random forests, have spread. These approaches allow to deal with complex interactions and nonlinearities in the prediction models. Additionally, they do not require preselection of predictors and can be used also when the number of covariates is larger than the number of observations [40, 41]. However, the performance of these methods is not well stated. It depends on the presence and relevance of possible interaction effects and on the correlation structure of the data, and it could be quite poor when data are highly skewed [42–44]. It would be interesting to investigate the robustness to departure from the MAR assumption for multiple imputations approaches based on recursive partitioning. However, at the best of our knowledge, there are no studies dealing with such algorithms under the MNAR assumption and extending the pattern-mixture model to this context deserves ad hoc investigation.

### Limitations

We fixed the number of imputations to 40 following a rule proposed by White and colleagues [26]. Since in this preliminary analysis we focused on different quantities, all surrogate in respect to the primary endpoint of the ACTION study, we did not perform a detailed investigation in order to determine an optimal number of imputations, which in principle should be based on the Fraction of Missing Information evaluated for the parameter of interest [26] or on criteria aimed to assure results stability over repetitions of the MI procedure [45].

The present results could be sensitive to the prediction models specification. Models with a larger number of predictors or with predictors selected with different methods (e.g. LASSO) or models which includes interactions could result more or less robust to violation of the MAR assumption, leading to different conclusions. This point will be addressed when the mixed-pattern approach proposed in this paper will be applied on the complete ACTION data set. As one of the main limitations of the present study, we would like to remark that we did not account for the cluster randomized design of the study, because information on treatment assignment and cluster variables (country and hospitals) was blinded to us [46, 47]. Similarly, also patient’s survival at follow up was not available, so that we had no information about possible truncation by death. At this stage of the study, truncation by death was likely a minor problem since the analyses have been carried out with a follow-up of 2.5 months from recruitment and most of the enrolled patients had a good WHO performance status at baseline (the median survival for both small and non-small cell lung cancer in both stage III and IV is estimated to be around 7–8 months [48, 49], and 2.6 and 1.7 years for patients with and without metastasectomy, respectively [50]). Truncation by death is however a very relevant point that should be address in the future in order to get reliable estimates of the treatment effect [51–54]. Moreover, the MI on the complete ACTION dataset will be performed separately by treatment arm [46].

## Conclusions

In imputing missing data in end-of-life care studies, sensitivity analyses for the departure from MAR should be performed. We proposed a modification of the MICE algorithm which accounts for the presence in the data set of two kind of missing data: missing entries satisfying the MAR assumption and missing entries, due to missing form, for which a MNAR mechanism could be hypothesised. We found that the results obtained after having imputed the missing values through MICE were not always robust to possible violations of the MAR assumption.

## Supplementary Information

**Additional file 1 Appendix.** Appendix with supplementary material

**Additional file 2.** ACTION study patient questionnaire. ACTION study patient questionnaire

## Data Availability

The datasets used and/or analysed during the current study are available from the corresponding author on reasonable request.

## Abbreviations

ACP: Advance care planning
AIC: Akaike’s information criterion
DY: Dyspnoea
EF: Emotional functioning
FA: Fatigue
MAR: The missing at random
MCAR: Missing completely at random
MI: Multiple imputation
MICE: Multivariate Imputation by Chained Equations
MNAR: Missing not at random
NARFCS: Not-At-Random Fully Conditional Specification
PA: Pain
PF: Physical functioning
QOL: Quality of life
REC: Research Ethics Committee
