# Supplementary material for: Missing not at random in end of life care studies: multiple imputation and sensitivity analysis on data from the ACTION study
Source: BMC Med Res Methodol. 2021 Jan 9;21:13. doi: 10.1186/s12874-020-01180-y (PMC7796568; doi:10.1186/s12874-020-01180-y)
Supplement: Supplementary file 1 — Additional file 1 Appendix. Appendix with supplementary material [file 12874_2020_1180_MOESM1_ESM.docx]

**Appendix**

**Figure A1** Pattern of missing values.


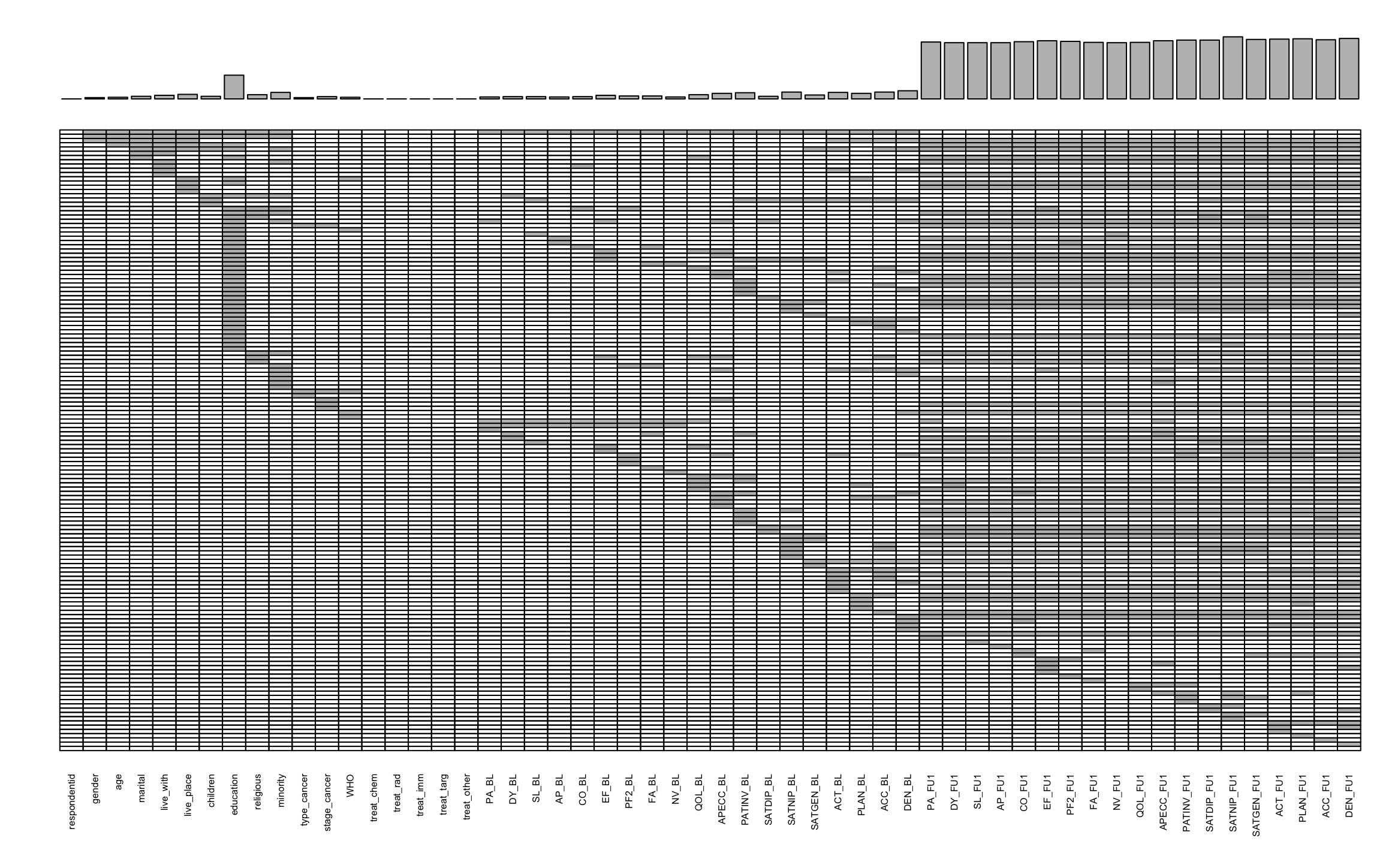


**Figure A2** Selected predictors used in the imputation procedure: for each row of the matrix, which indicates an incomplete variable, the columns marked with 1 represent the selected predictors (see Methods - Multiple imputation by Chained Equations under MAR assumption).

*
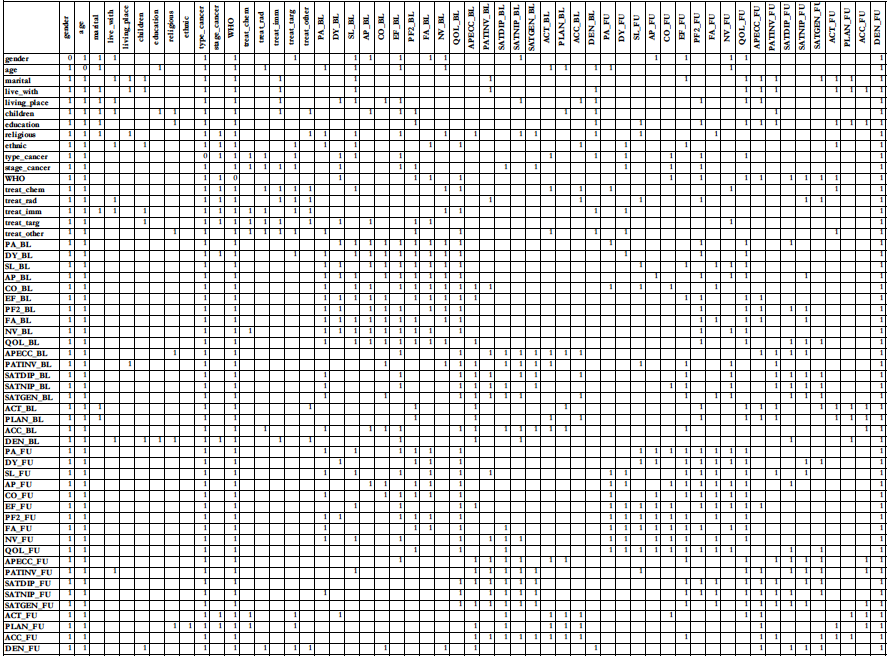
*

**Figure A3** Correlations (90% confidence intervals) between pain and the secondary endpoints (patient involvement, overall quality rating, active coping and denial) calculated after MI under the MAR assumption and under different MNAR models.


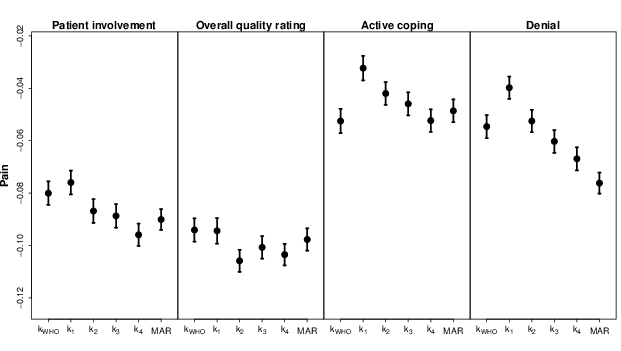


**Figure A4** Correlations (90% confidence intervals) between dyspnoea and the secondary endpoints (patient involvement, overall quality rating, active coping and denial) calculated after MI under the MAR assumption and under different MNAR models.

**
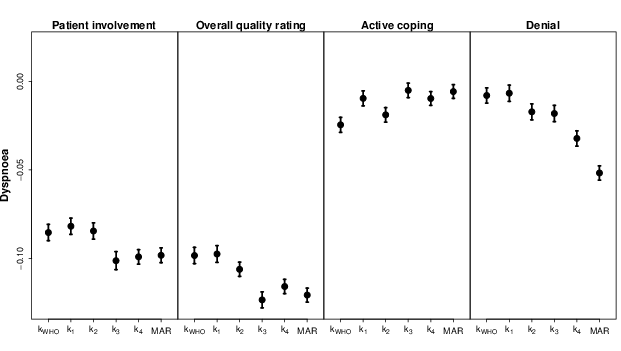
**

**Figure A5** Correlations (90% confidence intervals) between emotional functioning and the secondary outcomes (patient involvement, overall quality rating, active coping and denial) calculated after MI under the MAR assumption and under different MNAR models.

**
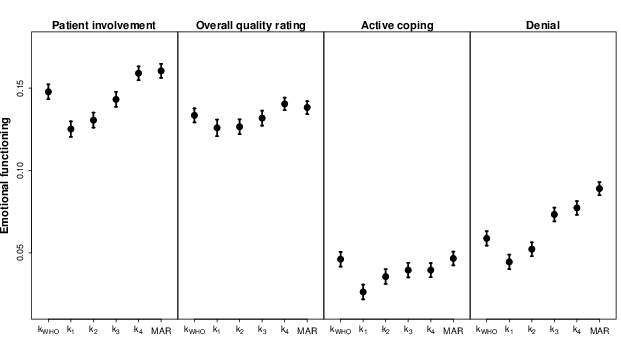
**

**Figure A6** Correlations (90% confidence intervals) between physical functioning and the secondary endpoints (patient involvement, overall quality rating, active coping and denial) calculated after MI under the MAR assumption and under different MNAR models.

**
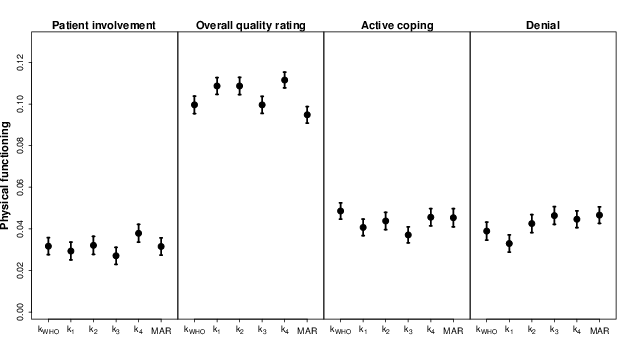
**

**Figure A7** Correlations (90% confidence intervals) between fatigue and the secondary endpoints (patient involvement, overall quality rating, active coping and denial) calculated after MI under the MAR assumption and under different MNAR models.


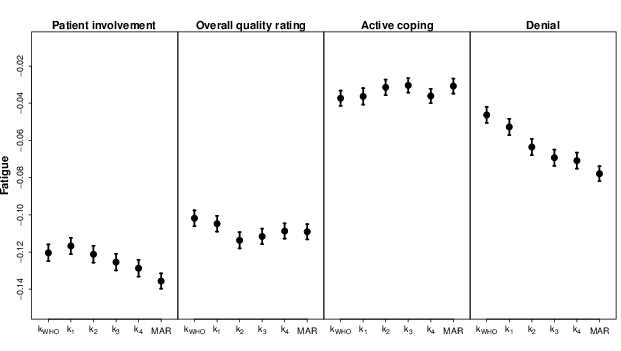


#### Table A1 Results of the sensitivity analysis using an approximation of the NARFCS approach (Tompsett et al., 2018) (see discussion): mean (90% confidence interval) of the observed and imputed scores at 2.5 months of follow-up under the MAR assumption and using different models for departure from MAR.

| **Variable** | **observed** | **k_WHO_** | **k_1_** | **k_2_** | **k_3_** | **k_4_** | **MAR** |
| --- | --- | --- | --- | --- | --- | --- | --- |
| Quality of life | 64.16 (64.09,64.24) | 57.27 (57.19,57.35) | 54.10 (54.01,54.18) | 56.13 (56.05,56.21) | 58.46 (58.39,58.54) | 60.74 (60.66,60.81) | 63.20 (63.13,63.27) |
| Pain | 20.67 (20.59,20.76) | 27.79 (27.68,27.89) | 33.93 (33.80,34.06) | 30.41 (30.29,30.52) | 27.44 (27.34,27.55) | 24.36 (24.27,24.46) | 22.32 (22.23,22.41) |
| Dyspnoea | 26.33 (26.23,26.42) | 36.41  (36.28, 36.54) | 43.53 (43.38,43.68) | 38.41 (38.28,38.54) | 33.89 (33.78,34.01) | 30.14 (30.04,30.24) | 27.03(26.94, 27.13) |
| Emotional functioning | 79.84 (79.77,79.91) | 73.33  (73.24, 73.43) | 65.9  (65.80, 66.03) | 70.03 (69.93,70.14) | 73.14 (73.05,73.23) | 76.19 (76.11,76.28) | 78.85 (78.78, 78.92) |
| Physical functioning | 68.69 (68.60,68.77) | 62.69 (62.60, 62.78) | 59.98 (59.89, 60.08) | 61.64 (61.55,61.73) | 63.49  (63.40,63.58) | 65.38 (65.29,65.47) | 67.44 (67.35,67.53) |
| Fatigue | 41.68 (41.59,41.76) | 50.88 (50.77,50.98) | 53.35 (53.24,53.46) | 50.45 (50.35,50.55) | 47.80 (47.71,47.90) | 45.17 (45.08,45.26) | 42.73 (42.64,42.82) |

#### Table A2 Results of the sensitivity analysis using an approximation of the NARFCS approach (Tompsett et al., 2018) (see discussion): mean (90% confidence interval) of the observed and imputed scores at 2.5 months of follow-up for males and females under the MAR assumption and under the MNAR model assuming a shift depending on the WHO score.

|  | **observed** | | **k_WHO_** | | **MAR** | |
| --- | --- | --- | --- | --- | --- | --- |
| **Variable** | **males** | **females** | **males** | **females** | **males** | **females** |
| Quality of life | 66.67 (66.60,66.74) | 60.06 (59.98,60.13) | 59.75 (59.68,59.82) | 53.49 (53.41,53.57) | 65.83 (65.76,65.90) | 59.20 (59.12,59.27) |
| Pain | 19.22 (19.15,19.30) | 22.92 (22.82,23.01) | 25.86 (25.76,25.95) | 30.72 (30.60,30.83) | 20.68 (20.59,20.76) | 24.83 (24.74,24.91) |
| Dyspnoea | 25.04 (24.95,25.14) | 28.69 (28.59,28.79) | 34.85 (34.75,34.95) | 38.78 (38.65,38.92) | 25.71 (25.62,25.80) | 29.04 (28.94,29.14) |
| Emotional functioning | 83.96 (84.02,83.90) | 73.02 (73.10,72.94) | 78.29(78.21,78.36) | 65.80 (65.70,65.90) | 83.11 (83.04,83.18) | 72.37 (72.30,72.45) |
| Physical functioning | 69.86 (69.77,69.95) | 66.50 (66.41,66.58) | 64.08 (64.00,64.16) | 60.59 (60.50,60.68) | 68.71 (68.63,68.79) | 65.51 (65.42,65.60) |
| Fatigue | 39.09 (39.01,39.18) | 46.19  (46.10,46.28) | 47.98 (47.89,48.07) | 55.28 (55.17,55.39) | 39.76 (39.67,39.85) | 47.25 (47.16,47.33) |
