# Supplementary material for: Missing not at random in end of life care studies: multiple imputation and sensitivity analysis on data from the ACTION study
Source: BMC Med Res Methodol. 2021 Jan 9;21:13. doi: 10.1186/s12874-020-01180-y (PMC7796568; doi:10.1186/s12874-020-01180-y)
Supplement: Supplementary file 2 — Additional file 2. ACTION study patient questionnaire. ACTION study patient questionnaire [file 12874_2020_1180_MOESM2_ESM.pdf]

ACTION study patient questionnaire

EORTC QLQ-C15-PAL +

We are interested in some things about you and your health. Please answer all of the questions yourself by circling the number that best applies to you. There are no "right" or "wrong" answers. The information that you provide will remain strictly confidential.

	Not at all	A little	Quite a bit	Very much
1. Do you have any trouble taking a <u>short</u> walk outside of the house?	1	2	3	4
2. Do you need to stay in bed or a chair during the day?	1	2	3	4
3. Do you need help with eating, dressing, washing yourself or using the toilet?	1	2	3	4

During the past week:

	Not at all	A little	Quite a bit	Very much
4. Were you short of breath?	1	2	3	4
5. Have you had pain?	1	2	3	4
6. Have you had trouble sleeping?	1	2	3	4
7. Have you felt weak?	1	2	3	4
8. Have you lacked appetite?	1	2	3	4
9. Have you felt nauseated?	1	2	3	4
10. Have you been constipated?	1	2	3	4
11. Were you tired?	1	2	3	4
12. Did pain interfere with your daily activities?	1	2	3	4
13. Did you feel tense?	1	2	3	4
14. Did you feel depressed?	1	2	3	4
15. Did you worry?	1	2	3	4

16. Did you feel irritable?	1	2	3	4

During the past week:

	Not at all	A little	Quite a bit	Very much
17. Have you felt panic?	1	2	3	4
18. Have you felt vulnerable?	1	2	3	4
19. Have you felt that nothing could cheer you up?	1	2	3	4
20. Have you felt miserable?	1	2	3	4
21. Have you felt desperate?	1	2	3	4
22. Have you been afraid of loosing control?	1	2	3	4

For the following question please circle the number between 1 and 7 that best applies to you:

23. How would you rate your overall <u>quality of life</u> during the past week?

1	2	3	4	5	6	7
Very poor						Excellent

APECC

If you and one of your doctors had to make any medical decisions about your treatment and care, how confident are you that you would be able to...

	Not at all confident	A little confident	Somewhat confident	Very confident	Completely confident
24. Take part in a detailed discussion with your doctor about the different available options	1	2	3	4	5

25. Let your doctor know if you had any concerns or questions about his or her recommendation	1	2	3	4	5
26. Tell your doctor about the option you would prefer	1	2	3	4	5
27. Work out any differences of opinion with your doctor, should they exist	1	2	3	4	5
28. Take responsibility for making the final decision	1	2	3	4	5

Below you will find some questions concerning your treatment and care. Please indicate whether or not you agree by circling the number that best applies to you.

	Strongly Disagree	Disagree	Neither agree nor disagree	Agree	Strongly Agree
29. I feel that my close relatives/close friends know what my wishes and preferences are in relation to my future treatment and care	1	2	3	4	5
30. I feel that my doctors know what my wishes and preferences are in relation to my future treatment and care	1	2	3	4	5
31. I feel that I am involved as much as I want to be when decisions are made about my future treatment and care	1	2	3	4	5
32. I feel that I have a great deal of influence on decisions in relation to my future treatment and care	1	2	3	4	5

We are interested in some things about you and your experience of the care received during the past two months. Please answer all the questions yourself by circling the number that best applies to you. There are no 'right' or 'wrong' answers. The information that you provide will remain strictly confidential.

During the past two months, how would you rate doctors, in terms of:

	Poor	Fair	Good	Very good	Excellent
33. The information they gave you about your illness?	1	2	3	4	5
34. The information they gave you about your medical tests?	1	2	3	4	5
35. The information they gave you about your treatment?	1	2	3	4	5

During the past two months, how would you rate nurses, in terms of:

	Poor	Fair	Good	Very good	Excellent
36. The information they gave you about your medical tests?	1	2	3	4	5
37. The information they gave you about your care?	1	2	3	4	5
38. The information they gave you about your treatment?	1	2	3	4	5

In general

	Poor	Fair	Good	Very good	Excellent
39. How would you rate the care received during the past two months?	1	2	3	4	5

If you have anything to add about the care you have received during the past two months, you are welcome to do so below:

There are many ways in which people react to stressful events. Please circle the number that best describes how you have been coping in relation to your *illness* during the past two months.

	I don't do this at all	I do this a little bit	I do this a medium amount	I do this a lot
40. I concentrate my efforts on doing something about my situation	1	2	3	4
41. I accept the reality of the fact that this has happened to me	1	2	3	4
42. I try to come up with a strategy about what to do in my situation	1	2	3	4
43. I take action to try to make my situation better	1	2	3	4
44. I act as though this hasn't even happened	1	2	3	4
45. I say to myself "this isn't real"	1	2	3	4
46. I pretend that this hasn't really happened to me	1	2	3	4
47. I learn to live with my situation	1	2	3	4
48. I think hard about what steps to take in my situation	1	2	3	4
49. I refuse to believe that this happened to me	1	2	3	4
50. I get used to the idea that this has happened to me	1	2	3	4
51. I accept that this has happened to me and that it can't be changed	1	2	3	4

The following questions ask about your personal status. This information is important as it provides background information about you and your current situation. Please tick the box that applies to your current situation.

52. Are you:

- ❑ Male
- ❑ Female

53. What is your age? _____ years

54. What is your present (official) marital status?

- ❑ Married/civil partnership
- ❑ Unmarried
- ❑ Divorced/separated
- ❑ Widowed

55. Do you live with a spouse/partner?

- ❑ Yes
- ❑ No

56. Do you live:

- ❑ In a private household
- ❑ In an institution/care facility
- ❑ Other
Please specify: _________________

57. Do you have children?

- ❑ Yes
- ❑ No

If applicable, number of children living at home with you: ____

58. What is your total number of years of education (including primary school, secondary school, further education college, vocational training and higher education etc.)? ____

59. Are you religious?

- ❑ Yes
- ❑ No
- ❑ Prefer not to specify

If yes, please specify your religious affiliation: ___________________

60. Do you consider yourself to be a member of a minority ethnic group in [name of your country]?

- ❑ Yes
- ❑ No

If yes, please specify: ___________________
